# Glut-1 explains the evolutionary advantage of the loss of endogenous vitamin C-synthesis: The electron transfer hypothesis

**DOI:** 10.1093/emph/eoz024

**Published:** 2019-08-28

**Authors:** Tabea C Hornung, Hans-Konrad Biesalski

**Affiliations:** Department of Nutrition, University of Hohenheim, Garbenstrasse 30, Stuttgart 70593, Germany

**Keywords:** glucose transporter, vitamin C, gulono-lactone-oxidase, vitamin C recycling

## Abstract

**Introduction:**

During evolution, some species including humans, monkeys and fruit bats lost the ability for ascorbic acid (AA) biosynthesis due to inactivation of the enzyme l-gulono-lactone oxidase (GLO) and subsequently became dependent on dietary vitamin C. There are four current hypotheses in relation to the benefit of vitamin C dependence in the context of adaptation and reproduction. Here we advance and test a new ‘electron transfer hypothesis’, which focusses on the role of the expression of glucose transporter 1 (Glut-1) in red blood cells (RBCs) in recycling vitamin C, thereby increasing the efficiency of micronutrient uptake.

**Methods:**

To evaluate the benefit of Glut-1 expression, we determined vitamin C uptake into RBCs and potential release from two different species, humans with l-Gulono-lactone-oxidase (GLO-loss) and pigs with functional GLO.

**Results:**

The oxidized form of vitamin C (dehydroascorbate, DHA) was transported into human RBCs via Glut-1. There was no transport of either the reduced (AA) or the oxidized vitamin in pig erythrocytes.

**Conclusion:**

We propose that the transport of vitamin C increases an intracellular electron pool, which transfers electrons from intracellular ascorbate to extracellular substances like ascorbyl free radical or DHA, resulting in 100-fold smaller daily requirement of this essential redox sensitive micronutrient. This would be an advantage during seasonal changes of the availability from food and may be the key for the survival of individuals without vitamin C biosynthesis.

**Lay Summary:**

40 million years ago some individuals lost the ability to synthesize vitamin C. Why did they survive such as humans until now? Individuals with a specific glucose transporter Glut-1 on their erythrocytes which transports vitamin C need less and are protected from scarcity due to seasons and food competitors.

## INTRODUCTION

Most animals are able to synthesize ascorbic acid (AA) from glucose in either the kidney or the liver [1]. About 61 million years ago, some mammals and primates, including our human ancestors, lost the ability for this endogenous vitamin C synthesis [2]. This occurred due to the inactivation of l-gulono-lactone oxidase (GLO) gene with the consequence that the last step of the ascorbate synthesis from glucose was blocked. From then on, these species, including some primates, guinea pigs and Indian fruit bats, have been dependent on dietary, daily intake of AA.

There is an ongoing discussion about the benefit of the inactivation of GLO and the selective pressures on this phenotype. There are four current hypotheses regarding the evolutionary advantage of the missing ascorbate biosynthesis [3]:

1. The ‘ascorbate-rich diet hypothesis’: This hypothesis tries to explain the inactivation of l-gulono-lactone through the presence of adequate vitamin C within the diet. In the habitat of our ancestors, abundant fruits containing ascorbate were available [4, 5]. Therefore, the GLO lacking species were adequately supplied with this micronutrient and antioxidant and did not need an endogenous synthesis.
2. The ‘ascorbate and fertility hypothesis’: This hypothesis proposes that older individuals required more vitamin C than younger individuals did. It is argued that when this essential substance became rare, older and less reproductive, individuals died and the younger and fitter ones survived and reproduced more successfully [6].
3. The ‘better electron ratio hypothesis’: During ascorbate biosynthesis, one ascorbate molecule is produced by consumption of glutathione (GSH) and by generation of one hydrogen peroxide molecule [7]. The net redox potential of this synthesis is electron neutral. Therefore, it was beneficial to pick up the required ascorbate by diet, which leads to an increase in the antioxidative capacity.
4. The ‘free radical’ hypothesis was postulated by Challem and Taylor in 1998 [8] They hypothesized that not a mutation but a retrovirus inactivated the GLO. The decreased ascorbate concentrations led to generation and accumulation of more radical substances attacking the DNA. This should led to a higher rate of molecular evolution due to DNA oxidation, and consequently enabling faster adaptation to environmental changes.

All these hypotheses discuss only the loss of the AA biosynthesis separately but not further special features of this loss. Indeed, all species which lost the vitamin C biosynthesis have a second common feature: they express an alternative glucose transporter (Glut) isoform in their red blood cell (RBC) membrane, Glut-1 [9]. Although the inactivation of the GLO occurred at different time periods over 100 million years of evolution, all these species share this particular characteristic of Glut-1 expression [4, 10]. Glut-1 may have played an additional role in RBCs regarding natural selection of the RBC-Glut-1 phenotype.

**Figure 1.**
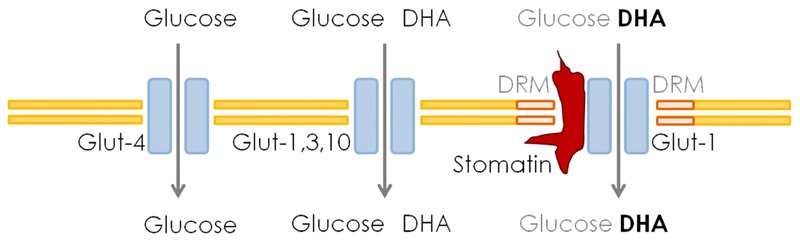
Schematic presentation of different glucose-transporters and their special features

Glucose transporters are passive carriers that enable the diffusion of glucose molecules via a concentration gradient [11]. To ensure the required energy uptake, intracellular glucose is phosphorylated by hexokinase to keep a positive concentration gradient into the cell. Glucose transport through Glut-1 is independent of insulin. However, Glut-4 is the most important insulin-regulated glucose transporter. Glut-4 is the regularly expressed isoform in mature mammalian RBCs and is responsible for their adequate glucose uptake [11]. RBCs do not need insulin for glucose transport, either from Glut-1 or Glut-4. Glut-1, the expressed isoform in mammalian species which lost the AA synthesis, transports glucose and dehydroascorbate (DHA), the oxidized form of vitamin C. Glut-1 has a second unique characteristic compared with the other DHA transporting Glut isoforms: Glut-1 can enter detergent resistant membranes (DRM) [12]. Within these DRM, the activity of Glut-1 can be separately regulated [13], a fact which compensates for the inability of insulin regulation (Fig. 1). This regulation is achieved by an integral protein called stomatin [14]. Stomatin is not only an anchor for cytoskeleton proteins as first suggested by Salzer and Prohaska in 2001 [14], it also interacts with erythrocyte Glut-1 resulting in a significant decrease of glucose uptake and an increase of DHA transport [9, 15]. After the loss of the ascorbate biosynthesis, the species were dependent on a continuous intake of vitamin C from food, otherwise, they would develop scurvy within a short time. No storage system for this water-soluble substance existed and the required amount was supposed to be 200–300 mg/kg of bodyweight (BW) per day [1].

AA is an essential cofactor for enzymes like copper-dependent monooxygenases or iron-dependent dioxygenases which are involved in dopamine and collagen synthesis, respectively [16]. Furthermore, ascorbate is the most important water-soluble antioxidant in plasma and in cells, where it acts as a primary antioxidant and interacts directly with radical species [17]. In membranes, it reduces alpha-tocopheroxyl radicals to alpha-tocopherol molecules and protects fatty acids from free radical induced peroxidation [18]. During radical neutralization, AA is oxidized once to ascorbyl free radical (AfR). This molecule can be oxidized further to DHA or two AfR molecules can dissociate to AA and DHA [19]. In physiological fluids like plasma, DHA is not a stable molecule and undergoes hydrolysis to 2,3-diketo-gulono acid with a half-life of 6–7 min and is eliminated by urinary excretion [20].

We propose a suite of three hypotheses to explain an evolutionary benefit of Glut-1 expression in context of the loss of vitamin C biosynthesis.

1. ‘Storage hypothesis’: Due to Glut-1 expression, the interaction with stomatin, and, consequently, the selective DHA transport into RBCs was enabled. RBCs reduce intracellular DHA and AA accumulates [21]. RBCs can act as vitamin C storage that takes up the oxidized instable form of vitamin C, protects it from degradation and renal elimination, and, releases the stored ascorbate pool back to the body fluids after RBCs undergo apoptosis. The storage of vitamin C may enable the compensation of fluctuating or decreased daily intakes. This theory is supported by narratives from sailors in the nineteenth century that described scurvy to develop after three months at sea [22, 23]. The maximum lifespan of human RBCs is 120 days, that is, after 120 days, all ‘vitamin C loaded’ RBCs underwent apoptosis and the ‘storage’ was empty [24]. To investigate this, the ascorbate content during RBCs' lifespan should be analysed. A corollary of this hypothesis is that older cells are predicted to contain more vitamin C than younger cells.
2. ‘Recycling hypothesis’: The expression of Glut-1 enables the selective and rapid uptake of DHA which is intracellularly reduced and ascorbate might be shuttled back to plasma. This would decrease the daily required amount and lead to a better survival in times of micronutrient scarcity. This is supported by findings from Mendiratta *et al.* [25], who report that RBCs are able to regenerate 35 µM ascorbate every 3 min *in vitro*. The recycling of DHA leads to a decreased daily requirement of this micronutrient via diet, because oxidized molecules are recycled, that is, reduced and are able to eliminate radicals again. A decreased requirement of vitamin C could have been a benefit in times when less fruits were available. We suppose an efflux or active transport of ascorbate out of the RBCs to assure antioxidant defense in the extracellular fluids.
3. Our last hypothesis we call the ‘electron transfer hypothesis’. The ability of RBCs to regenerate/reduce vitamin C *in vitro* depends on their intracellular redox system, the pentose phosphate pathway. But it is still unknown whether vitamin C, and especially ascorbate, can be disposed by RBCs. If neither Glut-1 as passive channel nor any other active transporter nor the apoptosis enables disposal of the accumulated reduced molecule, there must be another way to explain all the findings of vitamin C reduction and recycling [21, 25–27]. We propose that vitamin C could act as a kind of electron pool that stores electrons for cross membrane transport. This would increase the antioxidant defense immensely due to permanent and fast AfR reduction in blood.

In the following sections, we evaluate these three hypotheses experimentally.

## MATERIALS AND METHODS

### Blood withdrawals

All blood withdrawals from human volunteers were reviewed and approved by a local ethical committee (674/2013BO2). Blood was obtained from healthy volunteers aged between 24 and 46 years (*n* = 6). Blood from pigs was received from a local slaughter (*n* = 4). Blood was sampled in ethylenediaminetetraacetic acid-coated monovettes to prevent coagulation. Blood for vitamin C analyses was transported and stored in the dark on ice. The samples for erythrocyte separation were transported in the dark at room temperature and were immediately separated as subsequently described. About 18 ml of whole blood was withdrawn from each volunteer by a doctor. The whole blood was transferred to the top of a density layer (*ρ* = 1.081 g/ml) and centrifuged at 1000*×g* for 10 min. Plasma and buffy coat as well as the density layer were discarded and the RBCs were washed once with ice cold phosphate buffered saline (PBS) to remove residues of the density solution. Blood for vitamin C analysis was centrifuged at 1000*×g* for 10 min at 4°C. Plasma was stabilized with the same volume of ice cold 5% perchloric acid (PCA) for high performance liquid chromatography (HPLC).

### Uptake kinetics into RBCs

Isolated RBCs were incubated in PBS containing 3, 5 or 25 mM glucose and 100 µM ascorbate with or without ascorbate oxidase at room temperature. Ascorbate does not oxidize to DHA in PBS at room temperature (results not shown), so we did not work on ice to prevent RBCs from apoptosis by coldness. Every 15 or 30 min, a sample was taken, washed once with ice cold PBS and osmotically broken by addition of ultrapure water. Vitamin C was stabilized by the addition of 5% PCA (v:v 1:1) and ascorbate concentration was determined by HPLC as described below.

### RBCs separation by age

RBCs were isolated from whole blood as described above. For the differentiation of RBCs by age, three different density layers were used (*ρ* = 1.081, 1.092 and 1.098 g/ml). After centrifugation at 1000*×g* for 20 min at room temperature, the cells were carefully transferred into micro reaction tubes and washed with PBS once. The RBCs were aliquoted by 50 µl packed cells. One sample was used to determine the cell number with a Casy cell counting system. All other aliquots were osmotically lysed by adding 200 µl ultrapure water. For vitamin C stabilization, 250 µl 5% PCA was added to three samples and they were frozen at −80°C until HPLC analysis. A fourth sample was not stabilized with PCA but examined by photometric analysis to determine the haemoglobin content as well as the cell number.

### Efflux kinetics

RBCs were isolated from whole blood as described before. RBCs were ‘loaded’ by incubation in PBS with 5 mM glucose, 100 µM ascorbate and 5mU ascorbate oxidase/ml for 2 h adjusted to a haematocrit of 25% (2.5 ml packed RBC and 7.5 ml PBS). ‘Unloaded RBCs’ were incubated for the same way and time period in PBS with 5 mM glucose and 100 µM ascorbate. After incubation, cells were washed two times with PBS and then transferred into PBS (haematocrit 25%) containing only glucose. Samples of 400 µl (i.e. 100 µl packed erythrocytes and 300 µl PBS) were taken every 30 min. Intracellular and extracellular vitamin C concentrations were analysed by HPLC.

### Vitamin C determination

Samples were all stabilized 1:1 with 5% PCA and stored at −80°C until analysis. RBCs were lysed in ultrapure water 1:5 (v:v) before stabilization with PCA. Samples were thawed on ice and centrifuged (15 000×*g*, 5 min) to pellet proteins and cell organelles. The clear supernatant was transferred to an HPLC vial, protected from light and kept on ice. To reduce all forms of vitamin C (AfR and dehydroascorbic acid [DHA]), tris (2-carboxyethyl)phosphine (TCEP) was added to each sample, so that the complete vitamin C amount could be determined. The mobile phase was a 3.7 mM sodium dihydrogen phosphate buffer which was adjusted to pH 3.0 with meta-phosphoric acid. For sample separation, a reversed phase C18-aq column (Dr Maisch GmbH, Germany) with diameter of 5 µm was used. Detection occurred via electrochemical detection with a coulometric cell (analytic cell 2011A, esa). Concentration of the samples was interpolated via standards with LabSolutions software (Shimadzu Corp., Germany).

### Statistics

All data were tested for Gaussian distribution and homogeneity of variance. Data that did not meet these criteria were transformed before significance analyses. All statistics were made with SAS 9.4 (SAS Institute Inc.). To all datasets, a mixed model was adapted including qualitative and quantitative parameters. Individuals were taken as random effects. Degrees of freedom were adjusted by Kenward and Roger [28]. For all analysis, a *P*-value of <0.05 was considered to be significant and these data points are marked with asterisks.

## RESULTS

### Uptake of AA and DHA into human and pig RBCs

**Figure 2.**
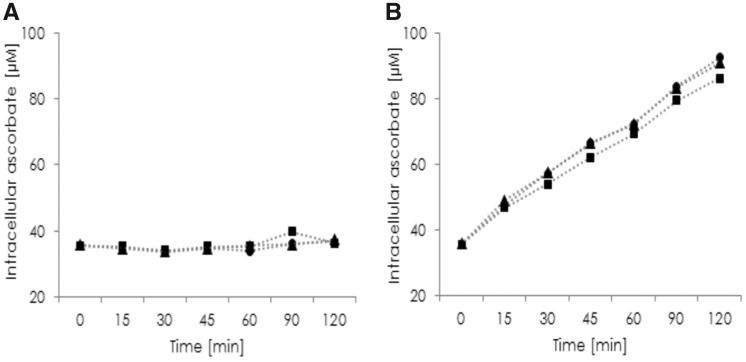
Intracellular ascorbate accumulation in human RBCs (*n* = 6) was determined over a period of 120 min. Cells were incubated in PBS containing 3 (circle), 5 (square) or 25 (triangle) mM glucose and 100 μM ascorbate (**A**) or 100 μM ascorbate with 5 mU ascorbate oxidase (**B**)

**Figure 3.**
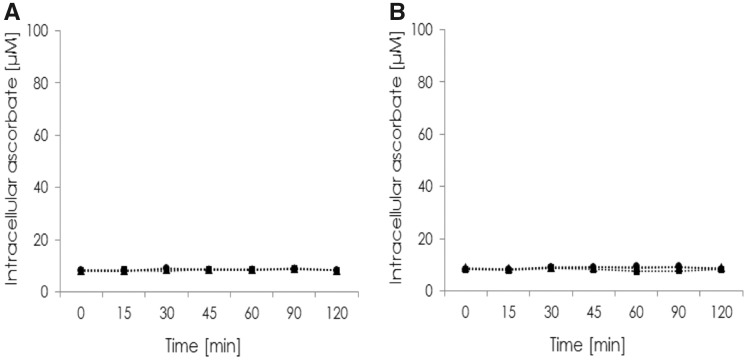
Intracellular ascorbate accumulation in pigs (*n* = 4) was determined over a period of 120 min. Cells were incubated in PBS containing 3 (circle), 5 (square) or 25 (triangle) mM glucose and 100 μM ascorbate (**A**) or 100 μM ascorbate with 5 mU ascorbate oxidase (**B**)

To verify that DHA transport into RBCs is significant and specific for species without vitamin C synthesis, uptake kinetics of AA and DHA into RBCs from pigs and humans were made. The results show that pig RBCs transport neither AA nor DHA (Fig. 2). The mean vitamin C concentration was 8.3 µM at the beginning of the kinetic. Human RBCs also do not transport AA, but intracellular ascorbate concentration significantly increases with DHA as Glut-1 substrate (Fig. 3). Mean intracellular AA concentration was 35.6 µM at baseline and increased to 89.8 µM. This demonstrates that human RBCs contain higher intra-erythrocyte vitamin C levels than pig erythrocytes.

### Vitamin content during RBCs lifespan

**Figure 4.**
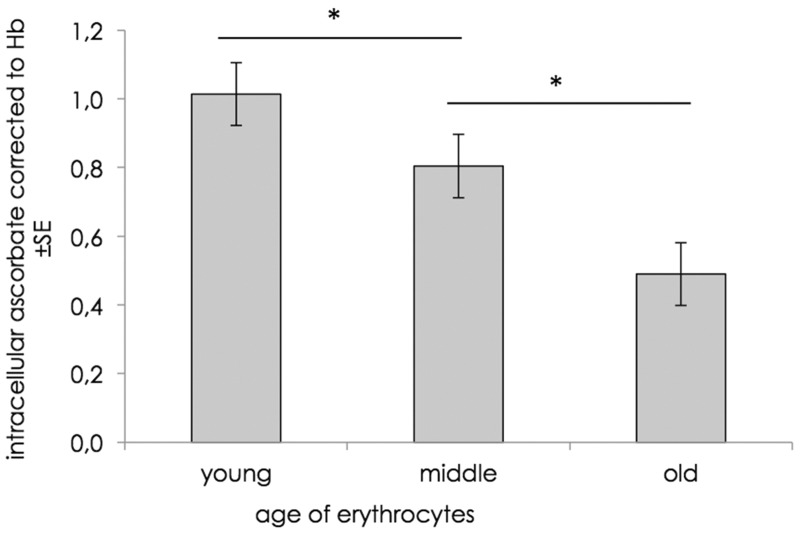
Intracellular ascorbate concentration of RBCs from six healthy volunteers that were separated by age before was corrected to the haemoglobin content. Significant differences (*P * < 0.05) of intracellular ascorbate concentration are denoted by asterisks

If RBCs may act as a kind of vitamin C storage, the micronutrient should accumulate during the lifetime of a cell. To verify the ‘storage hypothesis’ described above, RBCs from six healthy individuals were separated by their cell age. Three different density layers were used to isolate the 10% youngest and 10% oldest cells from all others (middle-aged cells). The intracellular ascorbate content was analysed by HPLC and was corrected to the haemoglobin absorbance (546 nm) and, therefore, the cell number. The results are shown in Fig. 4. Compared with middle-aged cells, youngest cells contain significantly more vitamin C and the oldest cells significantly less. The intracellular ascorbate content decreases during RBCs lifespan. Therefore, RBCs are no storage for vitamin C and do not accumulate the micronutrient over time until they undergo apoptosis.

### Efflux kinetics

**Figure 5.**
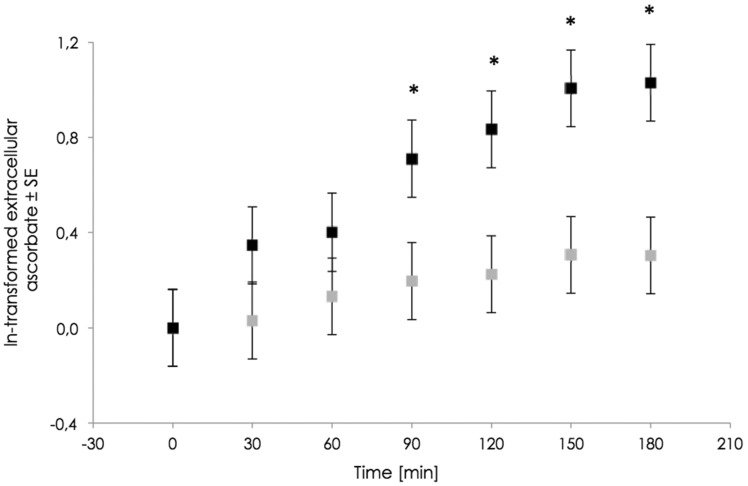
RBCs from three different volunteers were isolated and incubated in PBS containing 5 mM glucose, 100 μM ascorbate and with (black square) or without (grey square) ascorbate oxidase. After incubation for 2 h, cells were washed and transferred into PBS without vitamin C. Extracellular vitamin C accumulation (disposal from RBCs) was determined by HPLC analysis. Significant differences (*P* < 0.05) of ascorbate disposal are marked by asterisks

Figure 5 shows that intracellular ascorbate concentration decreases by time, that is, ascorbate must be released out of the cell. To verify the ‘recycling hypothesis’, RBCs from three different volunteers were incubated in PBS containing either DHA or ascorbate. After incubation, RBCs were transferred into PBS without vitamin C. The extracellular vitamin C concentration was determined every 30 min to observe a disposal of the micronutrient. After 90 min, loaded cells release significantly more vitamin C as unloaded RBCs.

There is a significant disposal of vitamin C from RBCs. Loaded cells release significantly more vitamin C than unloaded cells, but the absolute amount (1 and 0.5 µM) is negligible compared with intracellular concentration 89.8 and 36.5 µM, respectively. If there is an active transport of AA out of RBCs, the amount must be the same by loaded and unloaded cells. If the disposal of the antioxidant occurs by diffusion driven by a gradient, different released amounts were expected but on the whole, we would have expected a faster and, therefore, bigger amount that should have been found in the extracellular PBS. The results look more like a random, passive diffusion than directed transport. So the recycling hypothesis could not be confirmed by our findings.

## DISCUSSION

Our experimental results in relation to the uptake of DHA via the Glut-1 receptor, which only occurs in species that lost vitamin C synthesis, suggests a new hypothesis as to why the loss of vitamin C synthesis may represent an evolutionary advantage. We considered two other hypotheses. The electron transfer hypothesis shows that extracellular ascorbyl radicals (AfR), as produced during the redox defence reaction with other radicals, can be reduced again by electron transfer derived from DHA through the RBC membrane. As a consequence, AA is available again as redox partner in blood and interstitial fluid to inactivate free radicals. This is indeed a benefit because even at low levels of dietary vitamin C supply, this electron transfer helps to reduce the burden of free radicals. In contrast, the species not lacking GLO need far more vitamin C to ensure an equivalent redox defence reaction in the blood. We propose that this hypothesis offers a more complete explanation for the loss of vitamin C synthesis in primates and in some mammals.

### The various hypotheses do not justify the advantage of GLO inactivity

In the following sections, we summarize up our problems with these four theories:

The theory of ‘ubiquitous availability’ of ascorbate-rich food explains only why species without vitamin C biosynthesis may have survived in a vitamin C-rich habitat. It is frequently argued that all species which have lost the endogenous vitamin C synthesis have a vitamin C-rich diet. However, if a vitamin C-rich diet is a selective pressure for gene silencing, there should be far more species with an inactive GLO. Most species living in the environment of the last 100 million years experienced a diet rich in vitamin C, which is not only present in fruits, but in green leaves, nuts, beans, roots, seeds (e.g. seed coat baobab), insects and all kinds of small and large animals which consume a vitamin C containing diet. Consequently, it is not an advantage of frugivores and thus not a selective pressure.

The hypothesis of ascorbate as ‘fertility factor’ argues, that older creatures require more vitamin C and if they not get it will die and younger species with higher fertility will survive [6]. Primates live in family-like organizations. In times, when fruits and vegetables containing this essential micronutrient become scarce, prime-aged individuals may have even better access to resources through a combination of knowledge, physical ability and dominance rank.

The ‘electron ratio’ hypothesis discusses the advantage of oral vitamin C uptake versus biosynthesis regarding the net electron supply. The dietary intake of ascorbate has a positive electron ratio, whereas the synthesis is electron neutral. Further, the dietary intake of the micronutrient allows the organism to down regulate the synthesis and to use these glucose molecules for energy metabolism (glycolysis or glycogen synthesis). In an energetic view, this theory is plausible, but it does not explain why ‘dietary vitamin C dependent’ species are preferred to ‘independent’ species.

The most widely accepted hypothesis involves ‘free radicals’. Due to the loss of the ascorbate biosynthesis, more radical species accumulated in plasma and cells. Driven by more frequent DNA attacks, adaptation to environmental changes should be accelerated due to more frequent mutations. According to the proponents of this hypothesis, more frequent mutations should produce a greater diversity within one species and at least more or less successfully adapted to the changing environment. The most important dietary radical scavengers (carotenoids, vitamin E and vitamin C) are primarily present in plant-derived food. Based on this hypothesis, frugivores should have less mutations and consequently less phenotypic variability than carnivores with a poorer antioxidant supply.

All these hypotheses do not describe the real advantage of the loss of GLO and they fail to address the extraordinary aspect of Glut-1 expression in RBCs of the non-synthesizing species.

Glut-1 expression in RBCs—the common characteristic of all species without vitamin C synthesis—enables the energy independent and controlled transport of DHA into RBCs. The interaction of Glut-1 with stomatin results in a fast and selective transport of this molecule. We assume that this selective transport of vitamin C into RBCs is the reason for the selective pressure.

### Loss of GLO significantly reduces vitamin C requirement

To explain the advantage of the loss of vitamin C synthesis in connection with the occurrence of RBC-Glut-1, we have proposed three hypotheses.

First: RBC may act as a storage site for AA. Indeed, the uptake of DHA and subsequent conversion to AA prevents the irreversible breakdown and consequently the loss of this important vitamin. Our data however, do not justify this assumption because we do not find a significant accumulation of AA during the RBCs lifespan, which could increase blood ascorbate after erythrophagocytosis. Nevertheless, the uptake of vitamin C into RBC can contribute to a lower need for this vitamin through diet.

**Figure 6.**
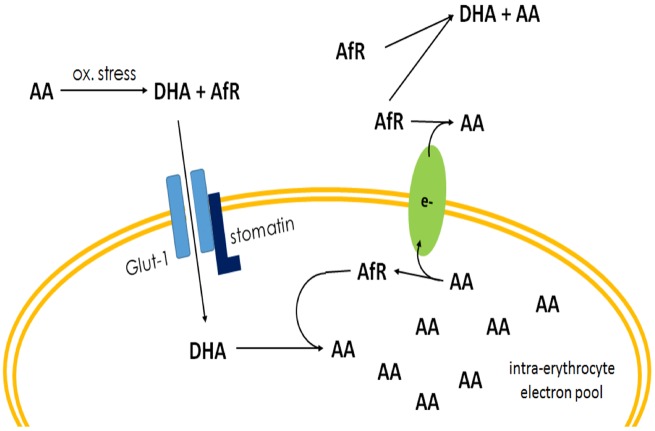
Oxidative stress and reactive oxygen species and radicals increase the production of ascorbate free radical (AfR) and DHA. DHA is transported by Glut-1/stomatin complex into RBCs where it is intracellularly reduced to AA. This electron pool is used to reduce extracellular AfR molecules and to prevent further production of DHA. The bigger the intracellular electron pool, the more effective is the recycling of extracellular vitamin C due to the independence of this pool from direct glucose uptake and hexose monophosphate pathway

Second: RBCs may act as a recycling compartment of vitamin C. Our results indicate that there is a passive diffusion of DHA via Glut-1 back to the extracellular fluids, which is relative to the intracellular vitamin C content. That means the reducing capacity as measured by Mendiratta *et al.* [26] cannot directly be based on vitamin C molecules which, as shown and justified by our results, are reduced and disposed back to plasma. Indeed, Van Duijn *et al.* [29] demonstrated that extracellular AfR molecules are recycled by an electron transfer over the erythrocyte membrane which supports our hypothesis. Electron donor was the intracellular accumulated AA. This explains not only the missing efflux of vitamin C in our *in vitro* measurements, but also the reducing capacity which was observed by Mendiratta *et al.* and this supports our ‘electron transfer hypothesis’ (Fig. 6).

Intracellular DHA reduction occurs directly or enzyme-dependent with GSH as electron donor [30, 31]. NADPH-dependent enzymes are also involved in DHA reduction, but GSH-dependent reduction is 10-fold more effective [26]. The reduction capacity of RBCs is not directly sensitive to Glut-1 inhibition by cytochalasin B [32]. This could be due to an intra-RBCs electron pool, as hypothesized, that enables extracellular reduction regardless of energy uptake and NADPH/GSH production.

As a consequence, the expression of Glut-1 might have had two advantages for individuals: First, DHA can be transported into RBCs quickly and is not degraded to diketogulonic acid which is excreted. Second, this intra-erythrocyte DHA accumulation and its reduction acts as an electron pool, which prevents the further production of DHA by AfR reduction. Due to DHA uptake via RBCs, Glut-1 individuals were able to ‘store’ more electrons for ‘cross membrane reduction’ than individuals expressing RBCs Glut-4. RBCs lose their nucleus during maturation and, subsequently, the ability for gene expression and protein biosynthesis. So, the capacity for electron storage by NADPH and GSH is limited. DHA uptake is the only opportunity to increase the electron pool. Third, the small amount of accumulated vitamin C might be shuttled back to the plasma, when RBCs undergo erythrophagocytosis or eryptosis and is available for radical neutralization again. To sum it up, the expression of Glut-1 enables efficient vitamin C recycling and decreases the daily required amount of this micronutrient significantly. Species without vitamin C synthesis require a minimum of 2–3 mg/kg BW and day [33]. It was calculated that species, with an active GLO, that is, erythrocyte Glut-4 expression, require up to 200–300 mg/kg BW per day [1]. To give an example, a 50 kg primate (Glut-4) has to consume 83–125 kg raw potatoes (an excellent vitamin C source) to reach the daily required vitamin C. A Glut-1 phenotype would need about 1 kg.

**Figure 7.**
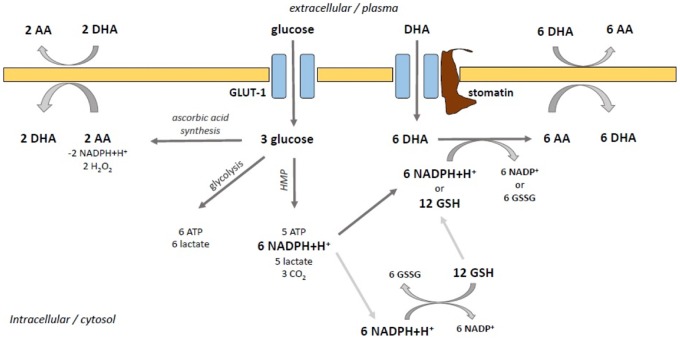
Comparison of the three different ways glucose can be metabolized: (1) Via anaerobe glycolysis: 6 energy equivalents (ATP) are produced. (2) Via the hexose monophosphate pathway (HMP): 5 Energy and 6 reduction equivalents are generated. (3) The ascorbate synthesis produces 2 AA from 3 glucose molecules, one glucose molecule is needed for ATP production (glycolysis) to activate the remaining two glucose molecules.

The recycling of extracellular ascorbate has two advantages: it maintains extracellular vitamin C by enhancing recycling of AfR to AA and it is energetically more efficient than the *de novo* synthesis of AA (Fig. 7): two AA molecules are produced from three glucose molecules. By metabolism of these three glucose molecules via the HMP, six NADPH and five ATP are built. That means 4 electrons on the side of AA synthesis versus 12 electrons on the side of HMP-based recycling. Including the minus of 2 NADPH on the side of AA synthesis and the fact that HMP is a cycle in which glucose is completely converted into NADPH with time, a net ratio of 0 electrons (AA synthesis) to 96 electrons (HMP recycling) results. This matches the observations, that species without ascorbate biosynthesis require up to 100-fold less ascorbate per day than synthesizing species and makes them relatively independent from vitamin C-rich food and its seasonal availability millions of years after the loss of the enzyme. And this indeed is an evolutionary benefit.

### Electron transfer capacity

All these facts support our third hypothesis, the electron transfer hypothesis. Van Dujin [29] showed *in vivo* that this electron transport indeed exists. To prove the electron transport *in vivo* is very difficult or nearly impossible. So we looked to diseases that are tightly linked to vitamin C depletion and/or increased oxidative stress (OS) like diabetes mellitus (DM). Based on preliminary data (unpublished), we found differences of intracellular vitamin C concentrations in RBCs. Further the distribution of Glut-1 and stomatin into DRM in patients with DM type 1 and their ratio show differences to controls despite equivalent plasma concentrations of both groups. There is an ongoing discussion on the real cause for secondary diseases in DM. Often lowered vitamin C plasma levels or increased OS is mentioned, but there is no evidence for one or both of these hypotheses due to contradictory results. If the expression of Glut-1 and its interaction with stomatin in RBCs was the key event for the compensation of the lacking vitamin C biosynthesis, a missing or disturbed protein interaction could also be the reason for the lowered VC plasma level and increased OS as described frequently in healthy and diseased individuals critically ill [34] and metabolic syndrome [35]. No or poor DHA uptake means a smaller intra-erythrocyte electron pool. This leads to less cross-membrane recycling, resulting in lowered vitamin C plasma level and, therefore, increasing OS. Indeed, in type 1 diabetes patients with low plasma vitamin C levels there was an association with adverse changes to microcirculation of peripheral arteries and a ventricular repolarization [36].

Sufficient uptake of DHA and conversion to AA into RBC might also contribute to a protection of RBC against OS. Incubation of RBC with vitamin C alone or in combination with N-acetylcysteine prevents the damage caused by OS in stored transfusion blood, such as increased osmotic fragility or membrane damage [37, 38]. More data are required to verify this assumption but our preliminary results show, that Glut-1 and stomatin ratio is an important factor in vitamin C metabolism in particular in DM patients.

### RBC-Glut-1 as a survival advantage

But still one question remains: Was induced GLO inactivation the selective pressure, leading to survival of RBCs Glut-1 positive individuals or was RBCs Glut-1 expression the benefit and the GLO-loss was just a consequence due to better energy balance?

Newborns of all species express in their first weeks of life Glut-1 in RBCs [10, 39]. In this time, the ascorbate biosynthesis is embryonically immature and not really working. This Glut-1 expression suggests that it is essential for adequate vitamin C supply in case of a missing synthesis.

In a population there are different phenotypes, that is, there might have been individuals with Glut-1 or Glut-3 or one or more of the other 12 Glut transporters in their RBCs. If the loss of GLO was caused by a retrovirus [8], this should quickly lead to vitamin C deficit in case of high vitamin C requirement of the previously synthesizing species, if they did not have Glut-1 on the RBC. The Glut-1-expressing individuals, however, had a clear advantage, as they needed much less dietary vitamin C. Based on our own data we can show, that in ODS-rats, a species which lost the GLO by a single nucleotide polymorphism, no Glut-1 is expressed in RBCs but Glut-3 is. The ODS rats need a fivefold increased amount of vitamin C compared to the Glut-1 species to ensure their requirement to be protected from severe vitamin C deficiency [40]. The synthesizing RBCs-Glut-4 expressing animals need the 20-fold amount compared with ODS rats and 100-fold amount compared with RBCs-Glut-1 phenotype [1, 40]. Not the general availability of vitamin C in fruits and other foods was an advantage for the non-synthesizing Glut-1 expressing individuals, but the fact that they were far less affected by fluctuations in vitamin C availability.

### GLUT-1 protects from dietary vitamin C shortage

It is argued [41] that the gene expressing the GLO became a pseudogene because its function was not further necessary due to a dietary environment rich in vitamin C. In the case of a missing disadvantage the pseudogenic allele could then be fixed by random genetic drift, without selective pressure against the gene loss [42]. If there is no selective pressure against the GLO gene loss because of the loss of the gene product and vitamin C is compensated via the diet, how can we justify the existence of the RBCs Glut-1? In these species (higher primates, guinea pigs and fruit bats), it is argued that gene loss may be a major motif of molecular evolution and an engine of evolutionary change [43]. The silencing of the GLO gene could favour individuals with another trait, the RBCs-Glut-1, which has been without visible advantage until the inactivation of the functional GLO gene and a potential scarcity of vitamin C due to seasonal differences in availability or food competitors. From this we propose that species with RBCs Glut-1 and active GLO might have lived together with species without RBCs Glut-1 and active GLO for a long time. Glut-1 expression is also a specific trait in frugivorous bats. But there is evidence that within the frugivorous bats, there are few with functional GLO genes (*Rousettus leschenaultii, Hipposideros armiger*) and others with expression of inactive GLO (*Pteropus vampyrus, Cynopterus sphinx* and *Rhinolophus ferrumequinum*) [43]. However, we do not know whether they express RBCs Glut-1 or only Glut-4. Regarding the selective pressure on Glut-1 it would be of interest to figure out whether these species, which are closely related and have a similar frugivorous and vitamin C-rich diet, but without active GLO, express a functional Glut-1 on their RBCs? The presence of Glut-1 in these species would be a strong argument for the selective pressure on individuals with RBCs-Glut-1 and contradict the hypothesis that the vitamin C-rich diet favours the loss of the GLO activity. Although the inactivation of the GLO occurred at different time periods during 100 million years, all these species share this particular characteristic of Glut-1 expression [4, 10]. Not the vitamin C-rich food favoured the phenotype with missing vitamin C synthesis, but the ability to compensate a vitamin C-poor diet with RBC-Glut-1.

### Outlook

This erythrocyte recycling capacity and AfR reduction should be further elucidated in diseases that are linked to increased radical production, like diabetes. The low levels of vitamin C in blood and RBC observed in diabetics may be the result of competition between high blood glucose levels and DHA intake via Glut-1 [44]. At the same time, less DHA is available due to low vitamin C blood levels. This and the hyperglycaemia thus lead to a reduced uptake of DHA into the RBC and consequently reduced extracellular recycling capacity, favouring OS. As a result, RBCs are also exposed to greater OS and thus lose their deformability, which is important for oxygen exchange [44]. Indeed, the burden of free radicals, OS, in diabetes is discussed as the major driver of chronic inflammation and diabetic comorbidities [45, 46].

More fragile and stiffer RBC are described in diabetics [47] and are held responsible for microvascular damage. Human RBC ascorbate concentrations show an inverse relationship to plasma glucose, osmotic fragility and severity of diabetes [44]. A more detailed analysis of the mechanisms leading to low plasma and RBC ascorbate levels in diabetics and its consequences could lead to new therapeutic approaches for the adequate supply of this important antioxidant. Further research could justify the frequently demanded higher vitamin C requirements in diabetics [48] and thus contribute to a better supply and subsequently to a reduction of co-morbidities and OS as recently described in diabetic rats [49]. Recently, it has been shown that adolescents with type 1 diabetes have a reduced RBC-Glut-1 [50]. This underlines the importance of an adequate supply of vitamin C in diabetic patients.

## LIMITATIONS

There are few methodological limitations: The separation of RBCs regarding their lifetime occurred by a density gradient at room temperature. Vitamin C content is influenced by light and temperature. Further, free haemoglobin possibly diminished the AA concentrations due to his oxidizing character. The absolute determined amount of vitamin C might have been decreased during this process. Out HPLC-method based on the indirect measurement of DHA and AfR, that is, only AA (the reduced form) is detected and DHA is calculated by the difference of whole AA (complete reduced samples with TCEP) to the stabilized sample without any reducing agents.

## CONCLUSION

Transport and accumulation of vitamin C into RBCs increases intra-RBCs electron pool and cross membrane electron transfer. This results in efficient extracellular recycling of vitamin C from AfR, produced during the redox reaction of vitamin C with free radicals. This recycling is energetically more economic compared with the *de novo* synthesis of the micronutrient. RBCs Glut-1 expression and resulting vitamin C recycling decreased the required daily amount by up to 100-fold and led to the evolutionary selection of this phenotype which is better adapted to a changing and unsecure supply of this important micronutrient.
